# Hybrid Stabilization of Kaolin Clay Using Biopolymer, Polypropylene Fiber, and Trivoltherm Waste: Mechanical Performance and Freeze–Thaw Durability

**DOI:** 10.3390/polym18101222

**Published:** 2026-05-17

**Authors:** Mehmet Uğur Yılmazoğlu, Bilge Aksu Alcan

**Affiliations:** 1 Department of Civil Engineering, Kastamonu University, Kastamonu 37150, Türkiye; 2 Department of Civil Engineering, Kafkas University, Kars 36100, Türkiye; bilge.aksu@kafkas.edu.tr

**Keywords:** kaolin clay, biopolymer stabilization, Xanthan Gum, polypropylene fiber, trivoltherm waste, freeze–thaw durability, hybrid stabilization

## Abstract

This study investigates the mechanical behavior and durability performance of kaolin clay stabilized using a hybrid system composed of Xanthan Gum biopolymer, polypropylene fibers, and Trivoltherm waste fibers. Experimental studies were designed according to the Taguchi L16 orthogonal array to evaluate the effects of different additive combinations. Unconfined compressive strength tests were performed after curing periods of 7, 28, and 90 days, while durability behavior was assessed through 5 and 10 freeze–thaw cycles. In addition, scanning electron microscopy analyses were conducted to investigate the microstructural characteristics of the stabilized soils. The results indicated that strength increased significantly with curing time, reaching a maximum value of 1186 kPa after 90 days. Statistical analyses showed that Xanthan Gum was the dominant parameter affecting strength development, contributing approximately 57–63% to the unconfined compressive strength behavior. Fiber additives also improved ductility, crack resistance, and freeze–thaw durability through reinforcement and crack-bridging mechanisms. The best-performing mixtures exhibited markedly lower strength losses under freeze–thaw conditions compared with untreated soil specimens. Analysis of variance results confirmed that the investigated parameters were statistically significant (*p* < 0.05), and the developed models showed high prediction accuracy (R^2^ > 85%). Overall, the findings demonstrate that the synergistic interaction between the biopolymer matrix and fiber reinforcement system provides an effective and sustainable hybrid stabilization approach for improving the engineering performance of clay soils.

## 1. Introduction

The rise in population and urbanization, together with the growth in industrial activities across the globe, has increased the necessity of construction works, particularly in regions containing cohesive soils having fine particle sizes. Fine-grained soils usually feature low strength, high compressibility, and poor drainage characteristics along with excessive settlement. For this reason, it is essential to enhance the geotechnical properties of such soils [1,2,3]. Therefore, improving the engineering properties of such soils has become a critical requirement for structural safety, service life, and economic sustainability [4,5].

To accomplish this task, several soil improvement technologies are available. However, soil stabilization can be considered one of the most efficient and commonly applied approaches for increasing the mechanical properties of weak soils [1,6,7]. The typical procedure of soil stabilization involves the use of chemical binders such as cement and lime. These materials demonstrate outstanding performance in boosting soil strength, decreasing compressibility, and increasing durability [8,9]. On the other hand, the production and application of these chemical binders are associated with high energy consumption and carbon dioxide emission. It has been reported that cement production leads to the release of about 7–8% of global CO_2_ emissions [10,11,12]. As a result of these problems associated with the environment, there has been a rapid rise in scientific research focusing on the development of eco-friendly and sustainable techniques for soil improvement [13,14,15,16,17]. It has been found that biopolymers are considered promising materials in view of their being eco-friendly due to being recyclable, biodegradable, and having low environmental impact. Literature states that polysaccharide biopolymers such as Xanthan Gum (XG) and Guar Gum (GG), among others, act as a gelling material in soils and improve soil strength by making bonds with soil particles and filling voids between them [18,19,20]. These materials form strong bonds within the soil matrix through physical and chemical interactions, including hydrogen bonding, electrostatic attraction, and gel network formation [21,22,23].

One of the commonly employed techniques for soil stabilization is through the introduction of fibers into the soil. Random fibers such as polypropylene, basalt, natural, and glass fibers offer tensile strength in the soil, restrain crack propagation, enhance stress distribution, and make the soil behavior ductile [24,25,26,27]. There is evidence showing that fiber-stabilized soils not only show improved mechanical properties but also have greater strength and stability under environmental impacts, including freeze–thaw cycles. Although freeze–thaw cycles can cause cracking and reduction in soil strength due to expansion and contraction effects, fibers prevent crack propagation, improving the behavior of the soil [28,29]. In this regard, polypropylene fibers are noteworthy. Among the commonly used synthetic fibers in soil stabilization techniques, polypropylene fibers are highly popular due to their low density, chemical inertness, strength, and economically feasible nature. Due to their hydrophobic property, they do not interact much with water, which is another reason why they are beneficial in terms of longevity. Polypropylene fibers randomly distributed in the soil, particularly under tensile loads, improve load transfer and hinder micro-cracking. Thus, the ductility of the soil is improved, whereas the brittle failure behavior is decreased along with the improved capability to absorb energy. Moreover, the frictional and adhesive interactions formed between the fiber and soil increase the strength of the soil as well. As mentioned in the literature, by adding polypropylene fibers, the strength reduction can be minimized, particularly in the case of frost–thaw cycles, and also preserve the stability of soil [30,31]. In addition to virgin synthetic fibers, recent studies have increasingly focused on recycled polypropylene-based reinforcement materials due to their environmental advantages and reduced carbon footprint. Shambilova et al. reported that recycled polypropylene modified with natural antioxidants such as Vitamin E can exhibit satisfactory rheological and mechanical properties while minimizing the environmental impacts associated with conventional synthetic stabilizers [32]. These findings indicate that sustainable fiber-based reinforcement systems have significant potential for future geotechnical applications. At the same time, from an environmental point of view, in recent years, there has been increasing attention paid to using waste fibers in addition to man-made fibers to improve soil mechanics. Not only does it decrease the impact on the environment, but it also enhances the physical performance of soil. These types of material influence the behavior of soil due to mechanisms such as crack bridging, load transfer, and soil-fiber interaction [33,34]. For instance, it has been shown that waste Kevlar fibers can increase the strength and durability of clay soils under frost-thaw cycles [35]. It becomes clear that different kinds of high-performance synthetic fibers have a similar effect on improving the soil properties. In this context, the role of Trivoltherm material, an insulation material used in industry and producing considerable waste during manufacturing, for soil stabilization remains unexplored. Trivoltherm is described in the literature and the technical documents of the manufacturer as a multilayer electrical insulation material made from polyester film, aramid paper, and nonwoven sheets [36]. Large amounts of waste are created when cutting and shaping are performed in industry; most of it is thrown away without recycling. Using this waste in soil stabilization in the form of fibers will enable providing tensile strength comparable to synthetic fibers and bridging cracks. Also, because of its multilayer structure, it will help achieve additional strength development due to increased mechanical interlocking and friction between soil particles. However, the effects of using Trivoltherm waste in soil stabilization, especially during freeze–thaw cycles, are understudied in literature, making it a new material for researchers.

Although numerous studies have investigated biopolymer-treated soils and fiber-reinforced soils separately, limited research has focused on hybrid systems combining biopolymers with both synthetic and industrial waste fibers. In particular, the combined use of Xanthan Gum, polypropylene fibers, and Trivoltherm waste fibers has not yet been investigated in the literature. Each component in this composite system was intentionally selected to provide a distinct stabilization mechanism. Xanthan Gum was used to improve interparticle bonding and pore filling through gel formation, polypropylene fibers were incorporated to enhance tensile resistance and ductility, and Trivoltherm waste fibers were introduced to improve mechanical interlocking while enabling the sustainable reuse of industrial waste materials [37,38]. Therefore, the present study aims to investigate whether the combined interaction of these components can create a synergistic stabilization mechanism capable of simultaneously improving strength, ductility, and freeze–thaw durability without requiring additional chemical stabilizers.

In hybrid soil stabilization systems, different additives contribute through complementary mechanisms rather than acting independently. In the present composite system, Xanthan Gum (XG) primarily acts as a bio-binder by forming a gel-like matrix that fills pore spaces and enhances interparticle adhesion. Polypropylene fibers (PPFs) contribute tensile resistance and ductility through crack-bridging and stress redistribution mechanisms, whereas Trivoltherm waste fibers (TVFs), due to their rough and multilayer surface morphology, improve mechanical interlocking and frictional resistance within the soil matrix. Therefore, the stabilization mechanism investigated in this study is governed not by a single additive alone, but by the synergistic interaction between biopolymer bonding and fiber reinforcement. This hybrid interaction is expected to improve not only strength development but also post-peak behavior and freeze–thaw durability.

In the present study, the effects of xanthan gum biopolymer at different proportions (0%, 0.5%, 1%, and 1.5%), polypropylene fibers at different proportions (0%, 0.5%, 1%, and 1.5%) and different lengths (6 and 12 mm), as well as Trivoltherm waste fibers, on kaolin clay were investigated. The experimental program was designed using the Taguchi L16 orthogonal array, and the effects of various additive combinations were evaluated. Unconfined compressive strength (UCS) tests were conducted on the samples at different curing periods (7, 28, and 90 days), and durability performance was examined by applying 5 and 10 cycles of freeze–thaw tests. In addition, SEM analyses were performed to elucidate the mechanisms of stabilization. This study aims to present a sustainable and innovative soil-improvement approach that uses biopolymers, synthetic fibers, and industrial waste materials.

## 2. Materials and Methods

### 2.1. Materials

**Figure 1:**
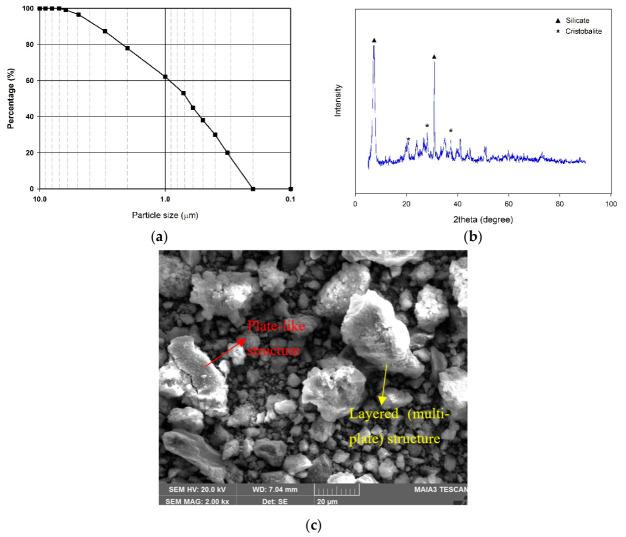
Characterization of kaolin clay: (**a**) particle size distribution, (**b**) XRD pattern, and (**c**) SEM image of raw kaolin clay.

In this investigation, the soil utilized is kaolin clay (KC) sourced from the province of Balıkesir in Turkey. Because of its fine grain size and plasticity, it is an appropriate type of soil for experimental purposes. The particle size distribution and XRD pattern of the soil used in the experiments are presented in Figure 1, while its physical properties, determined according to ASTM D4318-17 [39] standard, are shown in Table 1. It has been determined that the kaolin belongs to the low-plasticity clay (CL) class according to the Unified Soil Classification System (USCS). The natural water content and unit weight of the samples have also been determined. Additionally, the soil’s chemical composition was analyzed using X-ray fluorescence (XRF), and the results are given in Table 2.

The SEM image of the untreated kaolin clay revealed a plate-like and layered particle morphology typical of kaolinitic clay minerals. The presence of these structures indicates the presence of silicate and cristobalite, consistent with the XRD results. The particles exhibited relatively smooth surfaces and flake-shaped arrangements with localized particle aggregation. Such morphology contributes to the high surface activity and water retention behavior of kaolin clay and substantially influences the interaction between the soil matrix and stabilizing additives.

In this study, Xanthan Gum (XG), used as a biopolymer source, was obtained from a local company in Turkey. Xanthan Gum (XG) is a high-molecular-weight anionic polysaccharide produced by Xanthomonas campestris. Its molecular structure consists of a β-(1→4)-D-glucose backbone with trisaccharide side chains attached to alternate glucose units. Due to its hydrophilic and gel-forming nature, XG can form a viscous network structure within the soil matrix and improve interparticle bonding. Its density is approximately 1.5 g/cm^3^, and it rapidly dissolves in water to form highly viscous solutions. The rheological behavior of XG is pseudoplastic (shear-thinning), exhibiting high viscosity at low shear rates. It makes up the hydrogel phase in the soil, which fills up the soil pores by enhancing water-retention capacity and improving inter-particle binding force. Apart from that, the carboxyl groups create hydrogen bonding and electrostatic interactions within the soil matrix.

Polypropylene Fibers (PPFs) are used as a man-made reinforcing material. They were taken from a local company in Turkey and chosen in lengths of 6 mm and 12 mm, scattered in the soil sample. Some properties of polypropylene fibers are that they add tensile strength to soil, restrict crack development, enhance ductility, and improve stress distribution. Its density range lies in the range 0.90–0.92 g/cm^3^, tensile strength between 300–600 MPa, and modulus of elasticity in the range of 3–5 GPa. Elongation at break of fibers lies in the range of 10–25%, and due to its hydrophobic nature, water absorption capacity is very low (<0.1%). Chemically inert PPF is resistant to alkali and acid conditions.

**Figure 2:**
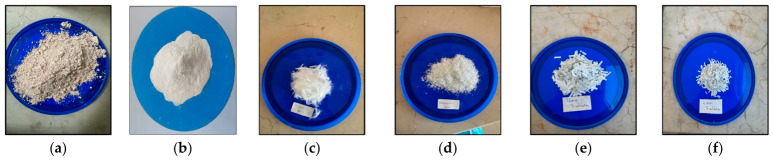
Materials used in the experimental study: (**a**) kaolin clay (KC), (**b**) Xanthan Gum (XG), (**c**) 12 mm polypropylene fibers (PPFs), (**d**) 6 mm polypropylene fibers (PPFs), (**e**) 12 mm Trivoltherm waste fibers (TVFs), and (**f**) 6 mm Trivoltherm waste fibers (TVFs).

Trivoltherm is an industrial insulation material that is commonly manufactured in sheet form. In this study, waste fragments generated during the cutting process were repurposed and used as reinforcement. These fragments were trimmed to approximate dimensions of 2 × 6 mm and 2 × 12 mm, allowing them to be incorporated into the soil mixture in a fiber-like form. Thanks to the multilayer, rough surface structure of the obtained Trivoltherm fibers (TVF), it is anticipated that they will create mechanical interlocking and bridging within the soil. Trivoltherm material has a multilayer structure comprising polyester film and aramid-based layers. The density of this material generally ranges between 1.2 and 1.4 g/cm^3^, and it exhibits high tensile strength and abrasion resistance. Additionally, due to its aramid content, it exhibits high thermal stability and maintains structural integrity under freeze–thaw conditions. The materials used within the scope of the presented study are shown in Figure 2.

### 2.2. Experimental Design and Sample Preparation

The experimental study was planned using the Taguchi L16 orthogonal array to examine the effects of multiple parameters with a minimum number of experiments. Within the scope of the Taguchi method, the effects of the factors were evaluated using signal-to-noise (S/N) ratios. The experimental design allows for the evaluation of the primary effects of the investigated parameters on soil behavior. The parameters (Table 3) considered in the experiments are, respectively: Trivoltherm fiber ratio (6 mm and 12 mm), polypropylene fiber ratio (6 mm and 12 mm), and biopolymer ratio. All additive ratios (Table 4) were expressed as percentages (%) of the dry soil weight. The additive dosage range (0–1.5%) selected for XG, PPF, and TVF was determined based on previous studies reported in the literature, together with preliminary observations regarding workability, homogeneity, and fiber dispersion within the soil matrix. Previous investigations have shown that excessive biopolymer or fiber contents may lead to agglomeration, insufficient mixing, discontinuous gel formation, and reduced mechanical efficiency. Therefore, a moderate additive range was selected to achieve balanced mechanical improvement while maintaining practical mixture preparation conditions. Although wider dosage ranges may provide additional optimization opportunities, this research primarily focused on evaluating the synergistic behavior of the hybrid stabilization system within practically applicable additive contents.

Before preparing the test samples, the soil was oven-dried at 105 °C for 24 h and sieved to remove coarse particles. The dried and sieved kaolin was subjected to the Standard Proctor test, and the required amount of water was added to reach the optimum moisture content obtained (%19.8). In the present study, the optimum moisture content (19.8%) of the untreated kaolin clay was maintained constant throughout the experimental program to provide a consistent basis for comparative evaluation of the investigated mixtures. Although the additives may influence the moisture demand of the soil, a constant moisture condition was adopted to isolate the effects of XG, PPF, and TVF on the mechanical and durability behavior of the stabilized mixtures. To ensure the homogeneous distribution of the biopolymer, the powder form of XG was dissolved in water and incorporated into the mixture. Afterward, polypropylene and Trivoltherm fibers were added to the plastic-consistency soil mixture and mixed until a homogeneous distribution was achieved. Once homogeneity was ensured, the samples were placed into the mold in three layers, with each layer compacted by applying equal energy. Within the scope of this study, samples with a diameter of 50 mm and a height of 100 mm were prepared using the static compaction method. After sample preparation, the test specimens placed in the molds were cured under laboratory conditions for 7, 28, and 90 days and then subjected to experimental procedures. Static compaction was adopted in the current work to provide uniform specimen preparation and repeatable laboratory conditions for comparative evaluation of the investigated mixtures. Although field compaction procedures may involve different loading mechanisms and energy levels depending on site conditions and construction techniques, the use of static compaction allowed consistent control of specimen density, moisture distribution, and additive dispersion throughout the experimental program. Therefore, the adopted preparation method was considered appropriate for evaluating the relative performance of the hybrid stabilization system under controlled laboratory conditions. The influence of field-scale compaction methods may be investigated in future studies.

### 2.3. Test Procedures

The UCS tests were performed following ASTM D1633-17 [40] to study the mechanical performance of stabilized soil specimens. During the tests, loading was applied under strain-controlled conditions at a constant deformation rate of 1 mm/min (approximately 1% strain rate). Samples were subjected to axial loading, while measurements of axial strain and load were taken continually. The maximum value of stress was used as the UCS of the sample. While performing tests, it is guaranteed that the sample ends’ surfaces remain uniform, and the load axis is aligned. The specimen dimensions used in this study (50 mm diameter × 100 mm height) were selected in accordance with standard UCS testing procedures commonly adopted in geotechnical laboratory investigations. The primary objective of the study was to comparatively evaluate the influence of different additive combinations under controlled laboratory conditions using a consistent specimen geometry. Although scale effects may influence the absolute mechanical behavior of larger specimens due to heterogeneity and fiber distribution variations, all mixtures in the present study were prepared and tested under identical conditions, allowing a reliable comparative assessment of the investigated parameters. The evaluation of scale-dependent behavior and larger specimen performance may be considered in future studies.

F-T tests were carried out to test the environmental durability of the soils under study. Samples were cured for 28 days, after which they were subjected to 5 or 10 F-T cycles. According to the requirements in ASTM D5918 [41], during every cycle, samples were cooled to −16 °C for 12 h before being exposed to room temperature for 12 h. Throughout the whole process, the water content in the samples was preserved to avoid any moisture loss.

SEM tests have been performed in order to investigate the microstructural features of stabilized soils. In SEM tests, the bonding characteristics of soil particles, pore characteristics, and interactions between the added materials and soil were analyzed based on SEM images. These tests provided important contributions to the stabilization mechanism. SEM tests have been performed by means of ZEISS scanning electron microscope at the MERLAB lab of Kastamonu University. Firstly, the tested samples were dried and then coated with gold/palladium to increase electrical conductivity. Different magnifications were performed in order to test the microstructural features of soil. The components observed in the SEM micrographs were identified based on their characteristic morphological features. Polypropylene fibers appeared as elongated and relatively smooth filamentous structures, whereas Trivoltherm waste fibers exhibited rough and layered surface textures due to their multilayer insulation structure. Gel-like regions surrounding soil particles were attributed to the Xanthan Gum matrix phase. Voids, crack formations, and fiber–matrix interaction zones were evaluated according to morphological continuity and surface characteristics observed in the SEM images.

In this study, the statistical analysis of parameters affecting the mechanical performance of stabilized soil samples has been performed by means of an analysis of variance (ANOVA) approach. As a part of statistical analysis, the effects of independent variables such as biopolymer amount (XG), polypropylene fibers ratio (PPF), Trivoltherm fibers ratio (TVF), and fiber length on unconfined compressive strength (UCS) have been investigated. ANOVA tests were performed under 95% confidence level (α = 0.05), and the parameters which had a *p*-value below 0.05 have been accepted as statistically significant. Effects of each parameter on UCS performance have been compared through F-value and contribution percentage. From this perspective, variables having higher F-values would be seen as having greater influence on the soil’s strength. The calculation of S/N ratios was carried out as one of the steps in the Taguchi method experiment. In order to maximize UCS values, the “larger-is-better” strategy was applied in the analysis. With this method, optimal combinations of different parameter levels were determined. ANOVA results, when compared, reveal the effects of experimental parameters on soil strength and enable the determination of the optimum mixture design. Additionally, the statistical findings obtained support the reliability of the experimental results and demonstrate the accuracy of the model. Statistical analyses were performed using the MINITAB software (Version 22.1). Using the main effects plots, S/N ratio graphs, and contribution percentages obtained from the analysis, the effects of the parameters on soil behavior were interpreted in detail. The statistical approach adopted in this study was designed to evaluate the dominant contribution of the investigated parameters while maintaining a practical experimental framework. In this research, the Taguchi L16 orthogonal array was primarily employed to identify the dominant effects of the investigated parameters on the mechanical and durability behavior of the stabilized mixtures while minimizing the required number of experiments. Due to the large number of factors and levels considered simultaneously, the analysis was limited to main effects in order to maintain an experimentally feasible design. Although interaction effects between parameters may also influence the stabilization behavior, evaluating all possible interactions would substantially increase the number of experimental combinations. Therefore, the present study focused on the primary factor contributions, whereas detailed interaction-based optimization may be considered in future investigations using advanced statistical approaches such as response surface methodology or full factorial design.

## 3. Results and Discussion

### 3.1. Microstructure Analysis (SEM)

**Figure 3:**
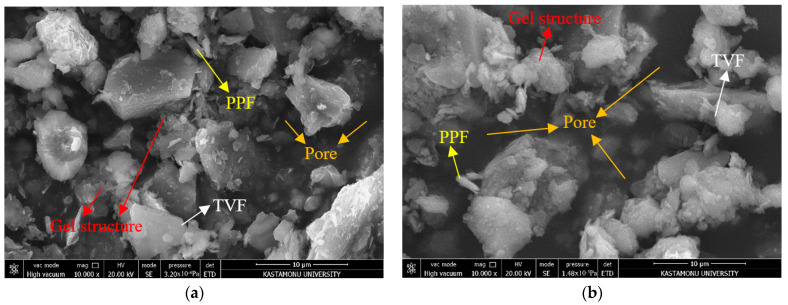
SEM micrographs of 28-day cured mixtures showing matrix continuity, gel-phase formation, fiber–matrix interaction, and pore structure: (**a**) M3 mixture and (**b**) M2 mixture.

SEM analyses were first evaluated to better understand the stabilization mechanisms and microstructural interactions within the hybrid system before discussing the mechanical and durability behavior of the mixtures. SEM images for the 28-day cured specimens are shown in Figure 3. For SEM examination, representative mixtures exhibiting relatively different mechanical behaviors after 28 days of curing were selected in order to evaluate differences in matrix continuity, pore structure, and fiber–matrix interaction. SEM micrographs of M3 mixture specimen (Figure 3a) show the presence of a relatively compact and well-integrated microstructure. The gel formation resulting from the 1% Xanthan Gum (XG) additive promoted the development of a more integrated and continuous matrix structure. In the M3 mixture, the fibers were homogeneously distributed within the matrix, and no distinct separation was observed at the fiber-matrix interface. Furthermore, the presence of relatively small and isolated voids suggests a reduced degree of interconnected porosity within the matrix. This integrated microstructure is consistent with the superior mechanical behavior observed in the M3 mixture. The SEM images of the M2 mixture (Figure 3b) point to a loose, discontinuous, and heterogeneous microstructure. The presence of distinct, in places interconnected, voids within the matrix indicates high porosity and weak inter-particle bonding. Additionally, the discontinuous and porous gel-like regions observed in the micrographs indicate that a low amount (0.5%) of XG could not form a homogeneous, dense network and that the gel phase did not develop adequately. Moreover, damage observed in the TVF fibers and the agglomeration of PPF fibers indicate insufficient fiber–soil adhesion and suggest that the fibers could not effectively participate in the load-transfer mechanism. The identification of fiber types within the SEM images was carried out according to their distinct surface morphology and geometrical characteristics. Polypropylene fibers generally exhibited smoother and more uniform filament structures, whereas Trivoltherm fibers displayed rougher and multilayer surfaces. These observations indicate that insufficient XG and fiber content limited gel continuity and reduced the effectiveness of the fiber-bridging mechanism. These microstructural features are consistent with the relatively low UCS behavior observed in the M2 mixture. The SEM findings obtained are consistent with the behavior of biopolymer-treated soils reported in the literature. Previous studies have stated that the presence of biopolymers contributes to the formation of a binding matrix between soil particles, filling the voids and enhancing adhesion by adhering to the fiber surface. Additionally, it has been reported that the bridging effect of fibers limits crack development [42].

**Figure 4:**
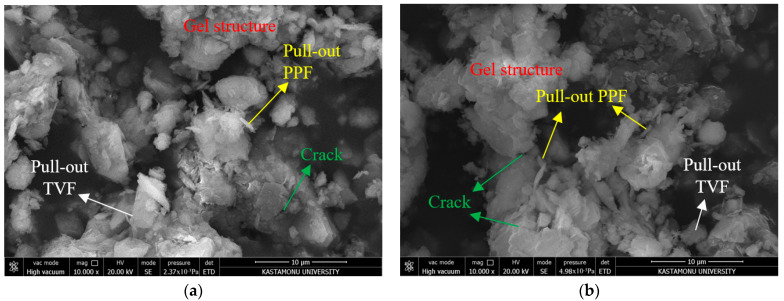
SEM micrographs of mixtures subjected to 10 freeze–thaw cycles showing crack propagation, matrix deterioration, and fiber–matrix interaction behavior: (**a**) M3 mixture and (**b**) M2 mixture.

To evaluate the durability-related microstructural response of the hybrid system, SEM images of specimens subjected to 10 freeze–thaw cycles are presented in Figure 4. Among the investigated mixtures, M3 and M2 were selected as representative samples to evaluate the influence of the hybrid stabilization system on microstructural durability under cyclic freezing and thawing conditions. The main reason for this is that in materials such as soil and concrete, mixtures exhibiting improved mechanical performance generally tend to possess more continuous and less porous microstructures. Composites with superior mechanical properties tend to exhibit lower strength loss under adverse physical conditions such as freeze–thaw cycles, whereas materials with lower strengths often show the opposite, as supported by numerous studies [43,44,45]. From the analysis of Figure 4a, it can be seen that the matrix structure of M3 remains well maintained, with the number and size of cracks being minimal. While there is partial debonding between the fiber and soil fabric for the two types of fiber materials (PPF and TVF), this damage is not prevalent, and the fibers still display crack-bridging properties in numerous locations. Additionally, although some microcracks are observed in the gel phase caused by the biopolymer (XG), this phase is not completely fragmented and largely maintains the inter-particle bonds. This relatively stable microstructure indicates that the hybrid stabilization system maintained matrix integrity and limited crack propagation even after repeated freeze–thaw exposure. In contrast, in the M2 mixture presented in Figure 4b, significant microstructural deterioration, increased cracking, the development of interconnected voids, and surface disintegration have been identified. Especially, the cracks, discontinuities, and layering observed in the gel structure indicate that the biopolymer network cannot provide sufficient resistance to the volumetric expansion occurring during freezing. The observed separations, void formations, and pull-out marks at the fiber–soil interface have limited the effective participation of fibers in the load-transfer mechanism. This contributed to the accumulation of internal stress within the matrix and accelerated crack propagation. Consequently, the deterioration of the structural continuity and fiber–soil interaction negatively affected the overall mechanical stability of the M2 mixture. Generally, appropriate amounts of biopolymer and adequate fiber reinforcement promote gel phase continuity, enhance the bonding between fibers and matrix, and minimize the initiation of cracks due to the freeze–thaw cycle. Based on the SEM results, the effects of biopolymer and fiber are evident from their influence on microstructure stability. Overall, the SEM observations suggest that the combined interaction of XG, PPF, and TVF contributed to improved microstructural continuity and crack resistance in the M3 mixture.

The SEM observations after freeze–thaw exposure further demonstrated the importance of biopolymer continuity and fiber reinforcement in preserving the integrity of the stabilized soil matrix. Mixtures containing sufficient XG and well-distributed fibers exhibited fewer interconnected voids, improved matrix continuity, and more effective crack-bridging behavior. The continuity of the gel phase and the effectiveness of fiber–matrix interaction appear to be critical parameters governing the durability behavior of the stabilized mixtures. In contrast, insufficient additive content resulted in discontinuous gel structures, fiber–matrix separation, and increased microstructural deterioration. These observations are consistent with previous studies reported in the literature [46,47,48].

### 3.2. Mechanical Properties

#### 3.2.1. Unconfined Compressive Strength (UCS) Results

**Figure 5:**
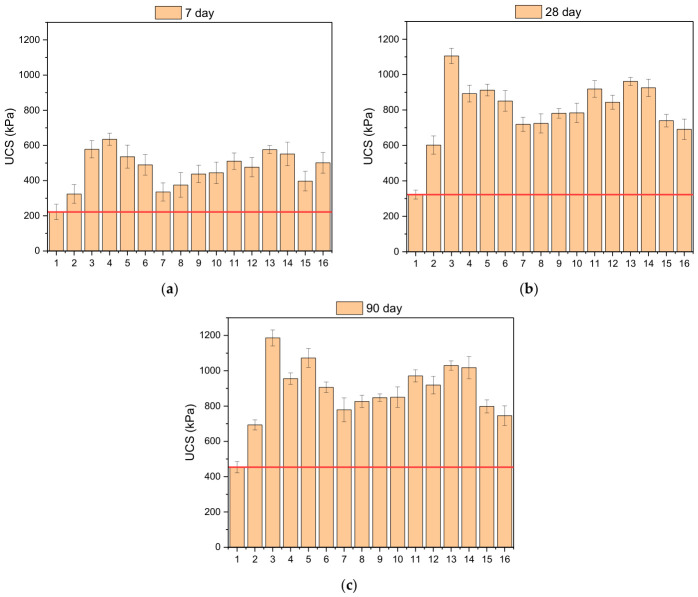
UCS values of the tested mixtures over several curing periods: (**a**) 7 days, (**b**) 28 days, (**c**) 90 days.

The unconfined compressive strength (UCS) results of the stabilized soil samples were evaluated for curing periods of 7, 28, and 90 days, as shown in Figure 5. The obtained results indicate that, for all mixtures, UCS values increased considerably with longer curing periods. UCS values, which ranged between 221–635 kPa after 7 days of curing, reached levels of 322–1105 kPa at the end of 28 days, and rose as high as 1186 kPa after 90 days of curing. These results demonstrate that the addition of biopolymer forms a more stable and stronger binding structure over time. Similarly, in the literature, Xanthan Gum (XG) has been found to form a hydrogel-like structure within the soil over time, significantly increasing interparticle bonding. This phase helps fill the pores, contributing to the development of a more continuous and integrated matrix structure. In addition, the addition of XG provides better interaction of the fibers with the matrix, improving their performance as well. This is believed to be one of the reasons behind the high strength and longevity of stabilized soils [21,49]. Considering the impact of the use of the additives—XG, PPF, TVF—on the UCS value, one can conclude that among the factors, XG is the most influential on the soil strength. This behavior is also supported by ANOVA analyses, where the impact of XG is assessed as comprising 57–63%. The action of XG in the soil is caused by the formation of hydrogels in addition to the cross-linking of the particles. The hydrogel phase fills the soil pores, improving matrix continuity and consequently enhancing strength behavior. It is known from the literature that biopolymers improve soil strength and provide other positive mechanical properties due to the formation of a gel network structure within the soil [50,51]. Although cement- and geopolymer-based stabilization systems may achieve higher UCS values in some applications, the primary objective of the present study was to develop a more sustainable and environmentally friendly stabilization approach using biopolymer and waste-based fiber reinforcement. The maximum UCS value of 1186 kPa obtained after 90 days indicates a significant improvement compared with untreated kaolin clay and falls within the strength range reported for several non-cementitious stabilization systems in the literature. In addition to strength enhancement, the proposed hybrid system also provided improved toughness, crack resistance, and freeze–thaw durability. In the present study, the primary focus was placed on UCS development, toughness behavior, and freeze–thaw durability of the stabilized mixtures. Future studies may further expand the mechanical characterization of the proposed hybrid stabilization system through modulus- and stiffness-based analyses.

Regarding the contribution of fibers, it can be noticed that polypropylene fibers contribute significantly to the soil behavior because, by adding tensile strength to the soil, they prevent cracking and ensure stress transfer. This happens due to the bridging action of fibers, and according to literature, fiber-reinforced soils are characterized by enhanced strength and ductility [47]. Even though the dosage of Trivoltherm fibers is relatively low, they enhance soil strength through mechanical interlocking due to their rough surface and layered nature. Such an observation emphasizes the significance of the cooperative contribution of fibers in the hybrid technique. After analyzing the findings, the M3 sample presented the best UCS results among the investigated mixtures at all curing periods. Due to the balanced combination of biopolymer and fiber additives, the M3 mixture developed an effective matrix structure and mechanical reinforcement for soil grains. Similar findings have been reported in the literature, where hybrid systems combining biopolymers and fibers demonstrated superior mechanical performance compared with individual additives used separately [52]. However, some mixtures, such as M15 and M16, exhibited relatively different long-term strength development trends. The different strength development trends observed in mixtures M15 and M16 may be associated with the complex interaction between fiber distribution and biopolymer matrix continuity. In mixtures containing relatively high fiber contents combined with limited or unbalanced XG content, homogeneous gel formation may become insufficient, resulting in localized voids and discontinuous bonding regions within the soil matrix. Furthermore, excessive fiber concentration may reduce particle contact efficiency and create weak interfaces due to fiber agglomeration. These effects can limit long-term stress transfer efficiency and lead to irregular strength development during curing periods. Similar behavior has also been reported in fiber-reinforced biopolymer-treated soils, where excessive fiber content may negatively affect matrix continuity and reduce long-term curing efficiency. In this hybrid system, xanthan gum (XG) acts as a binder that holds the soil particles together, polypropylene fibers (PPFs) provide tensile support, and Trivoltherm fibers (TVFs) contribute to better mechanical interlocking. As a result of this combined effect, the soil develops a more continuous and mechanically stable matrix structure. Although XG exhibited the highest statistical contribution to UCS development according to ANOVA results, the mechanical behavior observed in this study cannot be attributed solely to the biopolymer effect. The improved post-peak response, increased toughness, reduced crack propagation, and enhanced freeze–thaw resistance clearly indicate the contribution of fiber reinforcement mechanisms. In particular, polypropylene fibers provided tensile stress transfer and crack-bridging effects, while Trivoltherm waste fibers enhanced mechanical interlocking within the matrix. Therefore, the superior performance of the best-performing mixtures resulted from the combined and complementary interaction of biopolymer bonding and fiber reinforcement rather than from a single component alone.

#### 3.2.2. Load-Strain and Toughness Properties

**Figure 6:**
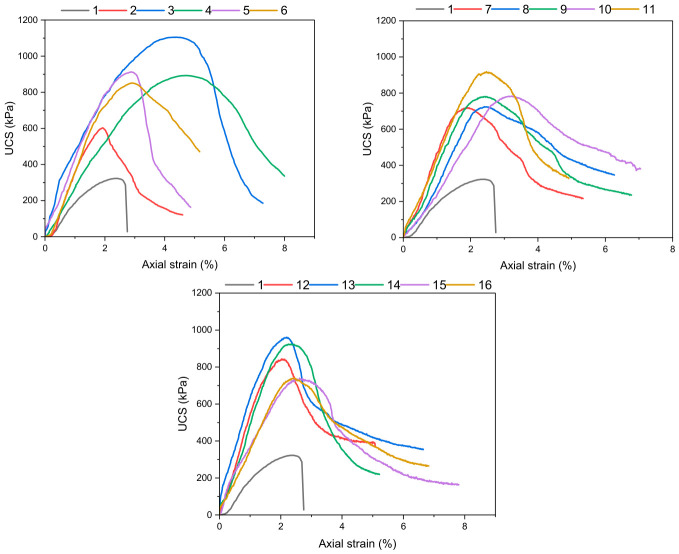
Stress–strain relationships for 16 distinct groups of specimens.

Other mechanical properties tested for the stabilized soils include the load-strain curve and toughness. According to the results obtained from the tests performed on these samples, biopolymer and fiber additives considerably affect the soil behavior. When analyzing the load-strain curve graphs for these samples, it is clear that the unstabilized samples showed a completely brittle behavior characterized by immediate loss of strength after maximum resistance (Figure 6). On the contrary, the soil samples having additive elements displayed much higher ductility with higher deformation capabilities even after fracturing, with clear residual strength. This means that there was considerable improvement in the behavior of this system after maximum resistance. Such a behavioral pattern can be attributed to the bridging action of the fiber additives. Fibers inhibit the growth of cracks, and this allows stresses to be evenly distributed, thus inhibiting the sudden breaking of soil samples. In addition, the binder matrix created by the biopolymer ensures efficient bridging action of the fibers [53,54].

**Figure 7:**
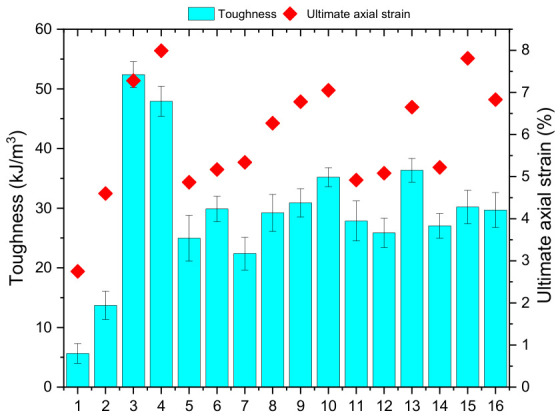
Variation in toughness and ultimate axial strain for the tested mixtures.

In cases where toughness (energy-absorbing ability) values are considered, it becomes evident that the presence of fiber reinforcement has a decisive impact on the soil properties (Figure 7). It has been confirmed that toughness values have shown a significant increase, particularly in the case of the mixtures that included polypropylene fibers. The highest toughness value has been found for the M3 mixture, thus confirming that these fibers improve energy-absorbing capabilities of the material and ensure energy distribution during deformation. It can be concluded from the results that biopolymer-fiber systems not only show improved strength characteristics but also deformation and energy dissipation properties. In the same way, the literature indicates that the incorporation of both biopolymers and fibers enhances soil adhesion and friction, leading to improved strength and deformation ability under applied forces [55]. This phenomenon was also seen in the present investigation, where the mechanical reinforcing effect of the fibers in tandem with the bonding effect of the biopolymer as a cohesive system was evident. In addition, it has been noted from the literature that soils reinforced with biopolymers alone may exhibit brittle characteristics, whereas their addition with fibers renders them ductile. This observation has been validated in this study.

The results suggest that the joint usage of biopolymer (XG), polypropylene fiber (PPF), and Trivoltherm fibers leads to the emergence of a hybrid mechanical behavior of soil. Under such a combination, the role of the biopolymer is binding and pore filling, while the fibers provide strength due to the presence of tensile resistance and crack regulation. Hence, such an interaction gives soil a more ductile structure and improves its durability.

### 3.3. Freeze–Thaw (F-T) Resistance

**Figure 8:**
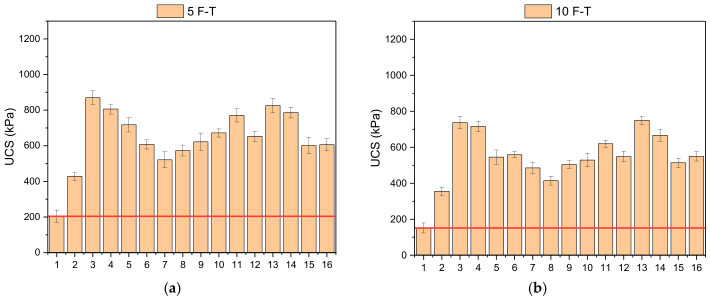
Variation in UCS results following different freeze–thaw periods: (**a**) 5 cycles, (**b**) 10 cycles.

Analyzing the results of the Freeze–Thaw (F-T) test, it can be stated that the strength of all samples decreased with an increase in the number of cycles. Nonetheless, this phenomenon was limited noticeably in cases when additives were present in soil samples (Figure 8). Based on the F-T test for five cycles, the UCS value varied between 204 and 870 kPa. While there was a dramatic decrease in the strength of the reference sample M1, the other samples kept their strength. For example, the strength value obtained in the M3 mixture was approximately 425% higher than that of the unmodified sample, indicating that the loss of strength was markedly limited. In the F–T results for 10 cycles, the UCS values ranged from 151 to 737 kPa. While the strength loss in the unmodified sample reached approximately 50%, it remained at 20–30% in the samples containing additives. Although a certain degree of strength reduction was observed after freeze–thaw exposure, the stabilized mixtures still maintained significantly higher strength levels compared with untreated kaolin clay, indicating improved durability performance under cyclic environmental conditions. This indicates that additive materials considerably increase soil durability against the effects of freeze–thaw cycles. The literature also states that soils with biopolymer additives are more resistant to freeze–thaw cycles and that strength losses are limited [56]. However, freeze–thaw cycles can also reduce soil strength over time. This is explained by microstructural deterioration caused by expansion during freezing and by changes during thawing [48].

**Figure 9:**
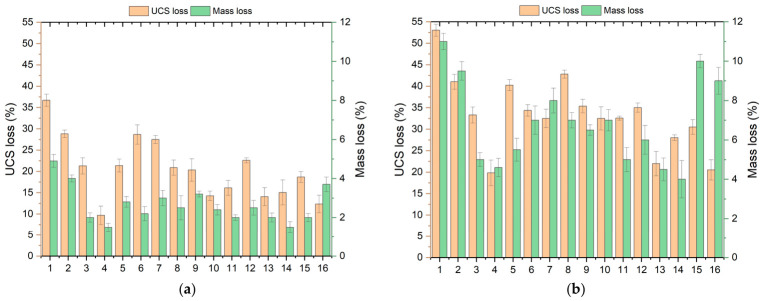
UCS and mass loss of samples after (**a**) 5 and (**b**) 10 freeze–thaw cycles.

When the loss of strength and mass after freeze–thaw cycles is evaluated together, it is observed that the biopolymer additive fills the voids within the soil, thereby limiting the harmful effects of water and reducing the loss of strength (Figure 9). The observation of higher mass loss and strength loss in samples with low XG content, in particular, demonstrates the critical role of the biopolymer in pore control. When the effects of fiber additives are examined, it has been determined that they restrict the progression of microcracks formed during freeze–thaw cycles and help maintain the integrity of the soil structure. Notably, the lower strength loss observed in samples containing fibers indicates that the fibers, through the crack-bridging mechanism, continue to transfer load.

In summary, the joint effect of using biopolymer and fiber as additives greatly enhances the soil’s resistance to freeze–thaw effects. In this composite approach, the biopolymer helps optimize the soil pore space and minimize water effects, whereas fibers help enhance mechanical strength and regulate cracking.

### 3.4. Statistical Analysis

In this study, the Taguchi method and analysis of variance (ANOVA) were used together to determine the effect of parameters on the mechanical behavior of stabilized soils. Additionally, the accuracy and reliability of the developed statistical models were evaluated using model summary parameters (Table 5). The analyses clearly revealed the relative effects of different parameters on soil performance. According to the ANOVA results, parameters with *p* < 0.05 were considered statistically significant. Upon examination, all main parameters (XG, PPF, and TRF) had a significant effect on UCS and freeze–thaw behavior. Moreover, high F-values confirm that the respective parameters have a strong effect on the model.

According to the unconfined compressive strength (UCS) results, biopolymer (XG) was the dominant parameter across all curing durations. According to the ANOVA results, the statistical contribution of the XG parameter to the variation in UCS values was approximately 62.7% for 7-day samples, 57.3% for 28-day samples, and 60.4% for 90-day samples. These contribution percentages do not represent the physical amount of XG within the mixtures; rather, they indicate the statistical influence of the XG parameter on the observed strength variation according to the ANOVA analysis. This suggests that the formation of a gel-supported structural framework due to biopolymer binding with soil particles noticeably enhances the bonding between soil particles. The development of such a continuous gel-supported matrix structure, which is filled with gel phase, is regarded as the principal factor contributing to the observed rise in soil strength properties. The effect of fibers on the mechanical properties of bio-soils was found to be significant for polypropylene fibers (PPFs) than for Trivoltherm fibers (TVFs). This demonstrates that polypropylene fibers improve the stress distribution within the soil more effectively due to their higher tensile strength and flexibility.

When examining the ANOVA results for the UCS values after freeze–thaw (F–T) test (Table 6), the biopolymer was also the most significant parameter affecting durability. According to the ANOVA analysis, the statistical contribution of XG to the variation in UCS values after freeze–thaw exposure was approximately 49.8% and 51.9% for 5 and 10 cycles, respectively. This finding indicates that the biopolymer improves the pore structure, thereby limiting the volumetric stresses caused by water during freezing. However, in the analysis of strength loss after the F-T test (Table 7), fiber additives played a more prominent role. The effects of polypropylene fibers (PPFs) and Trivoltherm fibers (TVFs) were evaluated both individually through ANOVA contribution analyses and collectively within the hybrid stabilization system. In particular, the effect of 6 mm Trivoltherm fibers on strength loss was determined to be 26–29%. This result shows that the fibers limit the propagation of microcracks formed during freeze–thaw cycles and help maintain the integrity of the soil structure. In the mass loss results after the F-T test (Table 8), the biopolymer was the dominant parameter. The ANOVA results indicated that XG was the dominant parameter affecting mass loss behavior, with statistical contribution values ranging between 45–66%.

**Figure 10:**
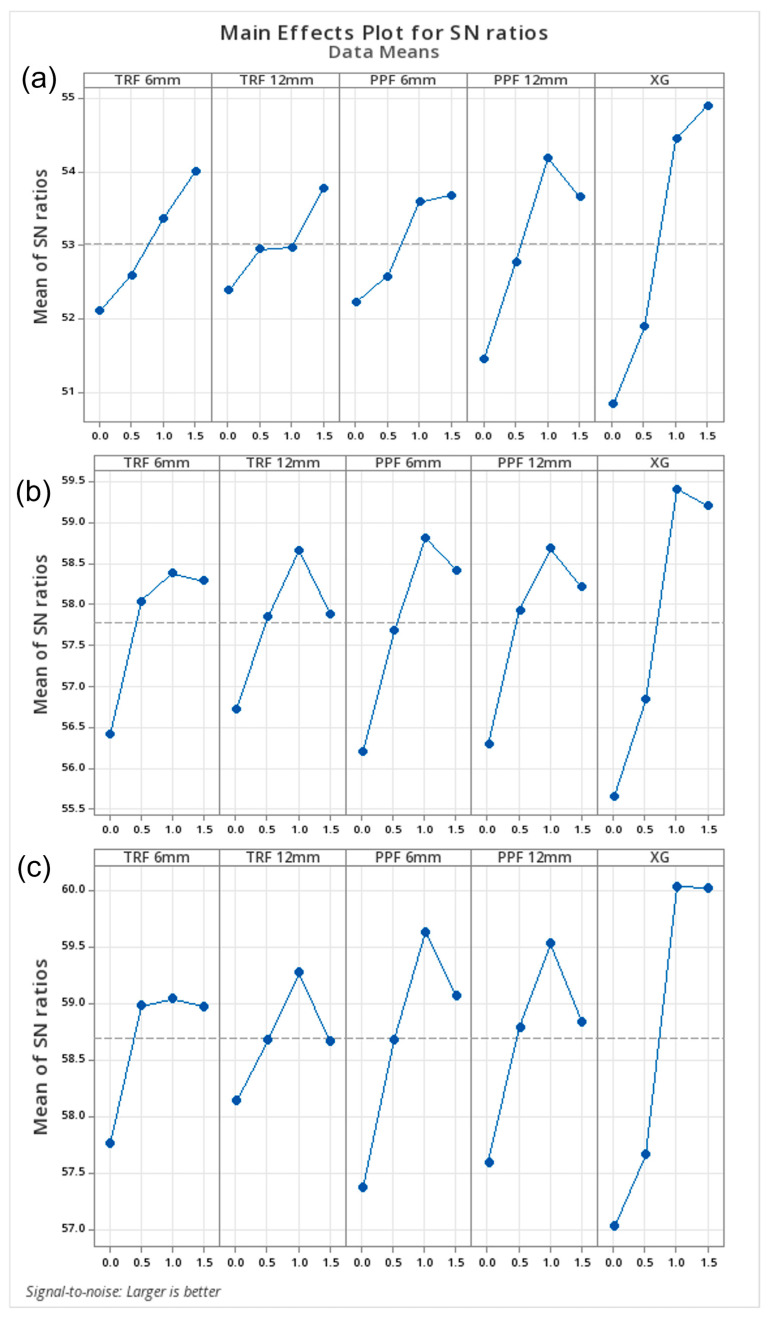
Taguchi S/N ratio response graphs for strength values after (**a**) 7, (**b**) 28, and (**c**) 90 days of curing.

The signal-to-noise (S/N) ratios obtained within the scope of Taguchi analyses are presented in Figure 10, and, according to the “larger-is-better” approach, optimal performance was achieved with balanced combinations of biopolymer and fiber additives. This result indicates that the hybrid stabilization approach provides superior performance compared to individual additives.

Upon analysis of the outcomes of the ANOVA together with those of the model parameters, it is clear that the models used are quite reliable. In the 7-day UCS model, the R^2^ value of 97.81% and the R^2^adj value of 96.60% indicate that the model fits the experimental data well. Similarly, in the 28-day model, the R^2^ was 94.15%, and in the 90-day model, 87.29%, indicating a limited decrease in model accuracy as the curing period increases. This situation is associated with the system behavior becoming more complex under long-term curing conditions. In the freeze–thaw models, very high accuracy values were also achieved. The R^2^ values of 97.18% and 98.05% in the 5 F–T and 10 F–T UCS models, respectively, indicate that this behavior is strongly explained by the parameters used. Additionally, R^2^pred values in the 93–95% range indicate that the model has high predictive power. In the strength loss models, however, the fact that the R^2^pred value for 10 F–T, in particular, drops to 56.95% indicates that system behavior becomes more irregular in advanced freeze–thaw cycles and that the model’s predictive performance decreases. Similarly, while high accuracy was achieved in the mass loss models for low cycles, the models’ performance decreased only slightly as the number of cycles increased. Previous studies in the literature support the statistical analysis evaluations conducted within the scope of this study [57,58,59].

Overall, when considering the ANOVA and Model Summary results together, it is clearly demonstrated that the combined use of biopolymer, polypropylene fiber, and Trivoltherm fibers creates a hybrid stabilization mechanism within the soil. In this case, the combination works such that, as the biopolymer acts as a binding medium, the fibers give mechanical stability to the composite, making it strong, durable, and flexible. The statistical analyses further support the synergistic behavior exhibited by the hybrid stabilization system in both interpretive and predictive terms.

## 4. Conclusions

In this study, the mechanical and durability properties of kaolin clay reinforced using biopolymer (XG), polypropylene fiber (PPF), and Trivoltherm waste fibers (TVFs) have been analyzed. The findings obtained are summarized below:

- It has been observed that with the increase in the curing period, the unconfined compressive strength (UCS) values in all mixtures increased significantly. The maximum UCS value was obtained as 1186 kPa at the end of 90 days.
- Biopolymer (XG) was the most dominant parameter throughout all curing periods, contributing approximately 57–63% to the UCS.
- It has been determined that polypropylene and Trivoltherm fibers have a significant effect on strength, especially in the medium and long term, and that the addition of fibers makes the soil behavior more ductile and increases its energy absorption capacity.
- As a result of freeze–thaw (F–T) tests, significant strength losses were observed in samples without biopolymer and fiber additives, while these losses were found to be considerably reduced in samples containing additives. In particular, it was observed that the strength loss in the mixtures with additives remained at around 20–30%.
- SEM analyses suggested that the continuity of the biopolymer matrix and the effectiveness of fiber–matrix interaction played important roles in governing the mechanical stability and freeze–thaw resistance of the stabilized mixtures. Mixtures exhibiting fewer interconnected voids and improved crack-bridging behavior generally demonstrated superior durability performance.
- As a result of the ANOVA analyses, it was determined that all parameters were statistically significant (*p* < 0.05) and that the biopolymer was confirmed as the most influential factor. The Model Summary results showed that the developed statistical models exhibited high accuracy (R^2^ > 85%) and reliability. Taguchi analyses revealed that optimal performance was achieved with balanced combinations of biopolymer and fiber additives.

Overall, the results demonstrated that stabilization was achieved through the synergistic interaction of biopolymer bonding and fiber reinforcement mechanisms rather than through the action of a single additive alone. In this hybrid system, Xanthan Gum provided pore filling and interparticle bonding through gel formation, while polypropylene and Trivoltherm fibers improved tensile resistance, crack control, and mechanical interlocking. The complementary interaction between these mechanisms enhanced strength, ductility, and freeze–thaw durability simultaneously. Therefore, the proposed composite stabilization approach offers a sustainable and mechanically efficient alternative for clay soil improvement.

## Figures and Tables

**Table 1:** Physical properties of the soil used in the experimental study [17].

Property	Value	Symbol and Unit
No 200#	66.5	FC (%)
Liquid limit	32	LL (%)
Plastic limit	24.8	PL (%)
Plasticity index	7.2	IP (%)
Specific gravity	2.54	G_s_
Clay ratio	19.9	C (%)
Silt ratio	46.6	M (%)
Sand ratio	33.5	S (%)
Natural moisture content	6.1	w (%)
Soil class: Low-Plasticity Clay (CL)		

**Table 2:** Chemical oxide compositions of soil.

Oxides (%)	Kaolin
CaO	0.15
SiO_2_	48.50
Al_2_O_3_	36.40
Fe_2_O_3_	0.80
MgO	0.10
TiO_2_	0.16
Na_2_O	0.15
K_2_O	0.20
Specific gravity	2.47

**Table 3:** Experimental parameters and their levels based on the Taguchi design.

Level	Parameter				
	6 mm TRF (%)	12 mm TRF (%)	6 mm PPF (%)	12 mm PPF (%)	XG (%)
1	0	0	0	0	0
2	0.5	0.5	0.5	0.5	0.5
3	1	1	1	1	1
4	1.5	1.5	1.5	1.5	1.5

**Table 4:** Experimental mixtures designed using the L16 Taguchi orthogonal array.

Mix No.	6 mm TRF (%)	12 mm TRF (%)	6 mm PPF (%)	12 mm PPF (%)	XG (%)
1	0	0	0	0	0
2	0	0.5	0.5	0.5	0.5
3	0	1	1	1	1
4	0	1.5	1.5	1.5	1.5
5	0.5	0	0.5	1	1.5
6	0.5	0.5	0	1.5	1
7	0.5	1	1.5	0	0.5
8	0.5	1.5	1	0.5	0
9	1	0	1	1.5	0.5
10	1	0.5	1.5	1	0
11	1	1	0	0.5	1.5
12	1	1.5	0.5	0	1
13	1.5	0	1.5	0.5	1
14	1.5	0.5	1	0	1.5
15	1.5	1	0.5	1.5	0
16	1.5	1.5	0	1	0.5

**Table 5:** Analysis of variance (ANOVA) of UCS values for samples cured for 7, 28, and 90 days.

7 day	Source	DF	Seq SS	Contribution	Adj SS	Adj MS	F-Value	*p*-Value
	TRF 6 mm	1	21,857	6.07%	21,857	21,857.4	55.44	0.000
	PPF 6 mm	1	25,661	7.13%	25,661	25,660.8	65.09	0.000
	TRF 12 mm	3	13,694	3.80%	13,694	4564.6	11.58	0.000
	PPF 12 mm	3	65,009	18.06%	65,009	21,669.6	54.96	0.000
	XG	3	225,822	62.74%	225,822	75,273.9	190.92	0.000
	Error	20	7885	2.19%	7885	394.3		
	Total	31	359,928	100%				
28 day	Source	DF	Seq SS	Contribution	Adj SS	Adj MS	F-Value	*p*-Value
	TRF 6 mm	1	42,643	4.54%	42,643	42,643	15.50	0.001
	PPF 6 mm	1	116,462	12.39%	116,462	116,462	42.33	0.000
	TRF 12 mm	3	66,492	7.07%	66,492	22,164	8.06	0.001
	PPF 12 mm	3	120,762	12.84%	120,762	40,254	14.63	0.000
	XG	3	538,870	57.31%	538,870	179,623	65.29	0.000
	Error	20	55,023	5.85%	55,023	2751		
	Total	31	940,253	100%				
90 day	Source	DF	Seq SS	Contribution	Adj SS	Adj MS	F-Value	*p*-Value
	TRF 6 mm	1	20,841	2.34%	20,841	20,841	4.24	0.051
	PPF 6 mm	1	100,862	11.33%	100,862	100,862	20.50	0.000
	PPF 12 mm	3	117,350	13.18%	117,350	39,117	7.95	0.001
	XG	3	537,891	60.43%	537,891	179,297	36.44	0.000
	Error	23	113,166	12.71%	113,166	4920		
	Total	31	890,110	100%				

**Table 6:** Analysis of variance (ANOVA) of UCS values after 5 and 10 freeze–thaw cycles.

5 F-T	Source	DF	Seq SS	Contribution	Adj SS	Adj MS	F-Value	*p*-Value
	TRF 6 mm	1	83,761	10.03%	83,761	83,761	71.03	0.000
	PPF 6 mm	1	139,742	16.73%	139,742	139,742	118.50	0.000
	TRF 12 mm	3	43,892	5.25%	43,892	14,631	12.41	0.000
	PPF 12 mm	3	128,528	15.38%	128,528	42,843	36.33	0.000
	XG	3	415,992	49.79%	415,992	138,664	117.58	0.000
	Error	20	23,586	2.82%	23,586	1179		
	Total	31	835,500	100%				
10 F-T	Source	DF	Seq SS	Contribution	Adj SS	Adj MS	F-Value	*p*-Value
	TRF 6 mm	1	77,381	11.29%	77,381	77,381	115.76	0.000
	PPF 6 mm	1	117,005	17.08%	117,005	117,005	175.03	0.000
	TRF 12 mm	3	44,518	6.50%	44,518	14,839	22.20	0.000
	PPF 12 mm	3	77,040	11.24%	77,040	25,680	38.41	0.000
	XG	3	355,879	51.94%	355,879	118,626	177.45	0.000
	Error	20	13,370	1.95%	13,370	668		
	Total	31	685,193	100%				

**Table 7:** Analysis of variance (ANOVA) of strength loss after 5 and 10 freeze–thaw cycles.

5 F-T	Source	DF	Seq SS	Contribution	Adj SS	Adj MS	F-Value	*p*-Value
	TRF 6 mm	1	449.08	29.23%	449.078	449.078	109.13	0.000
	PPF 6 mm	1	244.03	15.88%	244.032	244.032	59.30	0.000
	TRF 12 mm	3	203.34	13.24%	203.339	67.780	16.47	0.000
	PPF 12 mm	3	290.99	18.94%	290.987	96.996	23.57	0.000
	XG	3	266.61	17.35%	266.609	88.870	21.60	0.000
	Error	20	82.30	5.36%	82.304	4.115		
	Total	31	1536.350	100%				
10 F-T	Source	DF	Seq SS	Contribution	Adj SS	Adj MS	F-Value	*p*-Value
	TRF 6 mm	1	585.65	26.44%	585.652	585.652	32.03	0.000
	PPF 6 mm	1	293.57	13.25%	293.573	293.573	16.05	0.001
	TRF 12 mm	3	277.97	12.55%	277.967	92.656	5.07	0.009
	PPF 12 mm	3	240.22	10.84%	240.219	80.073	4.38	0.016
	XG	3	452.04	20.41%	452.037	150.679	8.24	0.001
	Error	20	365.71	16.51%	365.711	18.286		
	Total	31	2215.16	100%				

**Table 8:** Analysis of variance (ANOVA) of mass loss after 5 and 10 freeze–thaw cycles.

5 F-T	Source	DF	Seq SS	Contribution	Adj SS	Adj MS	F-Value	*p*-Value
	TRF 6 mm	1	2.5000	9.56%	2.5000	2.50000	91.07	0.000
	PPF 6 mm	1	4.7610	18.20%	4.7610	4.76100	173.44	0.000
	TRF 12 mm	3	4.1250	15.77%	4.1250	1.37500	50.09	0.000
	PPF 12 mm	3	2.3350	8.93%	2.3350	0.77833	28.35	0.000
	XG	3	11.8850	45.44%	11.8850	3.96167	144.32	0.000
	Error	20	0.5490	2.10%	0.5490	0.02745		
	Total	31	26.1550	100%				
10 F-T	Source	DF	Seq SS	Contribution	Adj SS	Adj MS	F-Value	*p*-Value
	TRF 6 mm	1	2.916	2.13%	2.9160	2.9160	4.47	0.044
	PPF 6 mm	1	25.921	18.95%	25.9210	25.9210	39.75	0.000
	XG	3	91.010	66.53%	91.0100	30.3367	46.53	0.000
	Error	26	16.953	12.39%	16.9530	0.6520		
	Total	31	136.800	100%				

## Data Availability

The original contributions presented in this study are included in the article. Further inquiries can be directed to the corresponding author.
